# Supplementary material for: Effect of Wettability and Adhesion Property of Solid Margins on Water Drainage
Source: Biomimetics (Basel). 2023 Feb 1;8(1):60. doi: 10.3390/biomimetics8010060 (PMC9944117; doi:10.3390/biomimetics8010060)
Supplement: Supplementary file 1 [file biomimetics-08-00060-s001.zip › biomimetics-2185989-SM/biomimetics-2185989-supplementary-final.pdf]

Effect of wettability and adhesion property of solid margins on water drainage

Can Gao[1,2]*, Lei Jiang*[1,2]*, and Zhichao Dong*[1,2*]

[1]CAS Key Laboratory of Bio-inspired Materials and Interfacial Science, Technical Institute of Physics and Chemistry, Chinese Academy of Sciences, Beijing 100190, China

[2]School of Future Technology, University of Chinese Academy of Sciences, Beijing 100049, China

Corresponding Authors**:** Zhichao Dong, E-mail: dongzhichao@mail.ipc.ac.cn

Supplementary information

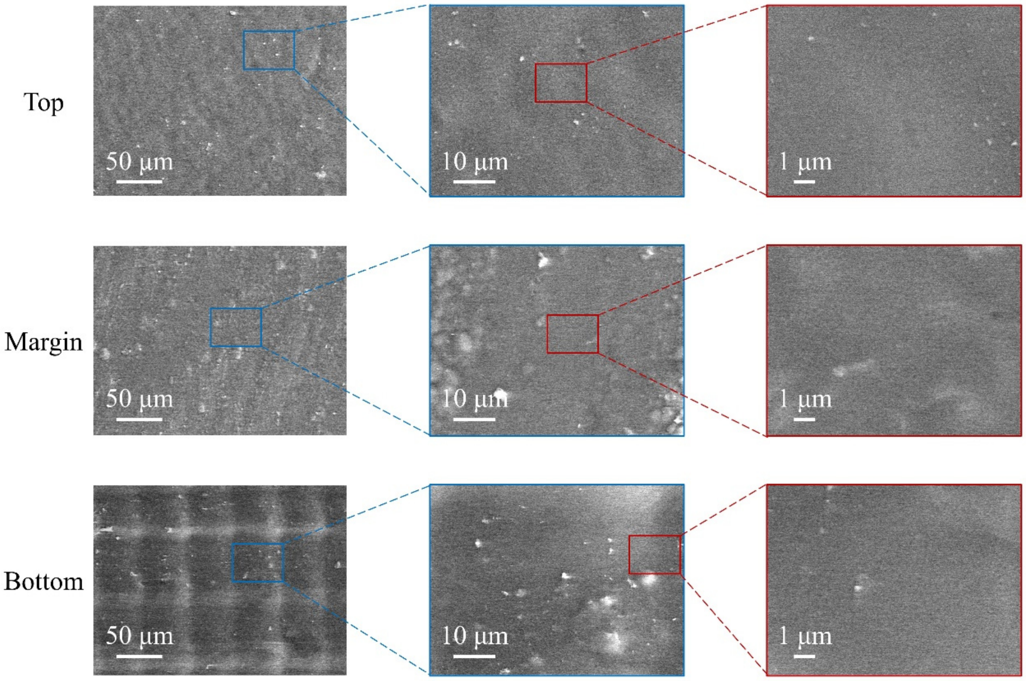

Figure S1. SEM images of top, front margin and bottom of the 3D-printed samples.

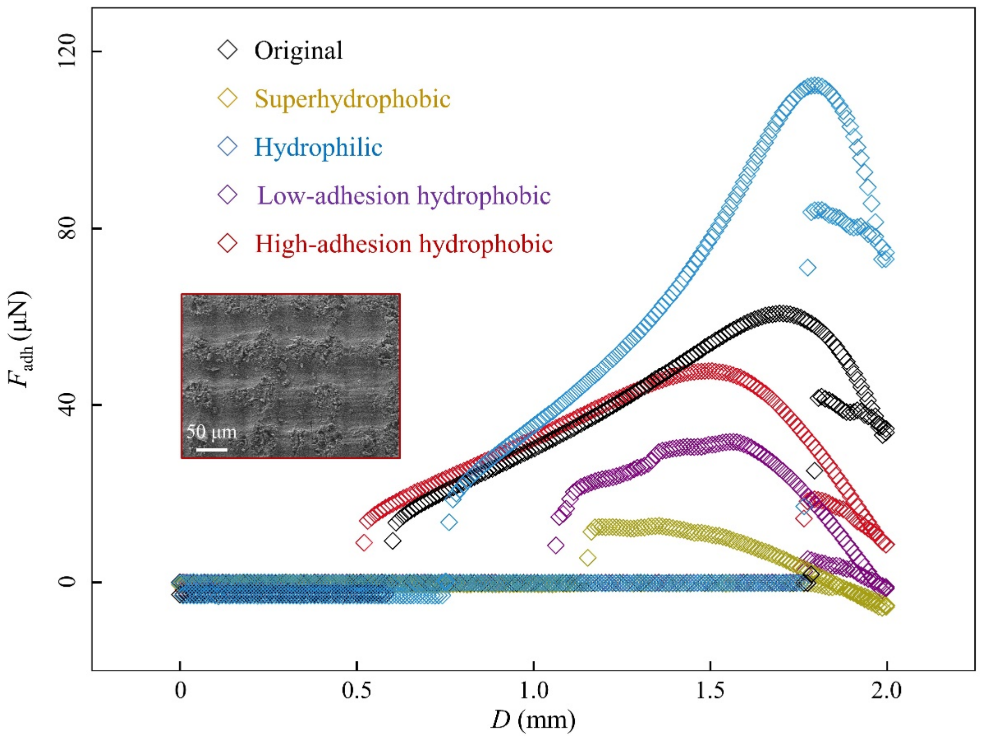

Figure S2. Adhesion forces of margin surfaces with different wettability modifications. (*inset*) SEM image of rubbed high-adhesion hydrophobic margin surface.

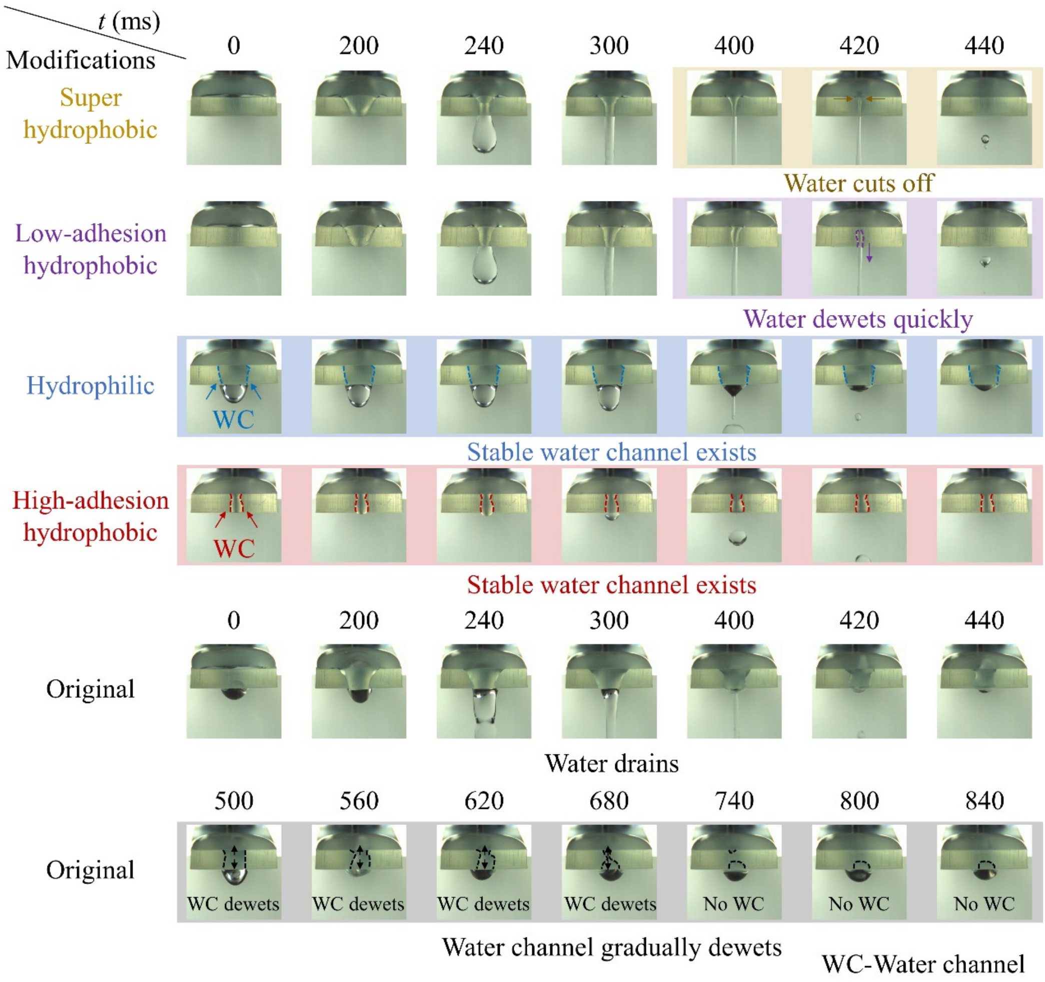

Figure S3. Selected drainage snapshots for samples of margins with different wettability modifications at Q = 5.0 mL/min from the front view.
